# Lessons from a Year of COVID-19 in Zambia: Reported Attendance and Mask Wearing at Large Gatherings in Rural Communities

**DOI:** 10.4269/ajtmh.22-0460

**Published:** 2022-12-12

**Authors:** Allison Juntunen, Jeanette L. Kaiser, Thandiwe Ngoma, Davidson H. Hamer, Günther Fink, Peter C. Rockers, Godfrey Biemba, Nancy A. Scott

**Affiliations:** ^1^ Department of Global Health, Boston University School of Public Health, Boston, Massachusetts;; ^2^ Right to Care Zambia, Lusaka, Zambia;; ^3^ Section of Infectious Diseases, Department of Medicine, Boston University School of Medicine, Boston, Massachusetts;; ^4^ National Emerging Infectious Diseases Laboratory, Boston University, Boston, Massachusetts;; ^5^ Center for Emerging Infectious Diseases Policy and Research, Boston University, Boston, Massachusetts;; ^6^ Household Economics and Health Systems Research Unit, Swiss Tropical and Public Health Institute, Basel, Switzerland;; ^7^ National Health Research Authority Pediatric Centre of Excellence, Lusaka, Zambia

## Abstract

Zambia instituted prevention behavior guidelines for social gatherings before the first case of COVID-19 was confirmed on March 18, 2020. Guidelines included nonpharmaceutical interventions (NPIs) including mask wearing, social distancing, and reducing sizes of gatherings. Within a larger cluster randomized trial of community-based parenting groups in four rural districts (three in Southern Province, one in Eastern Province), we collected 5,711 questionnaires from intervention participants between August 2020 and September 2021, during which the country saw two COVID-19 waves. Questionnaires asked about participation and behaviors at community gatherings. Generally, perception of risk of contracting COVID-19 was low for respondents in districts in Southern Province but higher for those in Eastern Province. The highest compliance to mask wearing was reported at clinics (84%) and church services (81%), which were the most frequently attended gatherings. Many funerals were attended by 200 to 300 people, but individuals were 30% less likely to report wearing masks (odds ratio [OR] = 0.71, 95% confidence ratio [CI]: 0.6–0.8) than those attending a clinic visit. After controlling for other variables, the odds of self-reported mask wearing at events were higher in January to March 2021 (adjusted OR = 1.5, 95% CI: 1.3, 1.7) and July and September of 2021 (adjusted OR = 3.0, 95% CI: 2.5–3.5), timepoints that broadly overlay with two COVID-19 peaks observed in Zambia. Results suggest guideline dissemination penetrated the rural areas. However, there is need to optimize the messaging to increase compliance to NPIs at high-risk gatherings, including funerals. The findings from this analysis should be considered as the COVID-19 pandemic continues to evolve.

## INTRODUCTION

COVID-19, a potentially severe, life-threatening respiratory illness caused by the SARS-CoV-2 virus, spread rapidly around the world in 2020, with the pandemic continuing today. The primary mode of virus transmission is via infectious aerosols generated when infected individuals exhale, speak, sing, cough, and sneeze.^1^ Consequently, nonpharmaceutical interventions (NPIs) for prevention have focused on facial coverings in addition to other NPIs such as shelter at home, closing schools and businesses, prohibition of public gatherings, and social distancing.

With improved understanding of SARS-CoV-2 modes of transmission and classification of high-risk activities, it is increasingly clear that “superspreading” events with large crowds, particularly those occurring indoors and when face masks are not worn, facilitate transmission.^2^^–^^6^ Events featuring activities that produce larger numbers of respiratory droplets and aerosols, such as singing, shouting, and loud speaking, are of particular concern. Choir practice has been documented as one example of a superspreading event where close proximity, poor ventilation, and increased circulation of SARS-CoV-2 droplets and aerosols from singing likely led to the infection of many other participants.^7^ The impact of a single superspreading event often extends well beyond those infected at the event with ongoing community spread leading to transmission to many more people over subsequent weeks.^8^ Identifying high-risk settings and drivers of superspreading events is crucial to COVID-19 control.

Like nearly all countries around the world, Zambia, located in southern Africa, has been adversely affected by the COVID-19 pandemic. Since the first case was confirmed in Zambia on March 18, 2020,^9^^,^^10^ the country has experienced four waves of COVID-19: an initial wave beginning in July 2020, a much larger second wave during January and February 2021, an even larger third wave during June and July 2021, and a fourth wave during December 2021 and January 2022. The second wave was most likely fueled by the South African variant of the virus (B.1.351, beta), which was first observed in Zambia in December 2020.^11^ As of June 24, 2022, there had been more than 324,922 confirmed cases and 4,000 deaths from COVID-19 in the country.^12^ Additionally, the epidemic may be worse in Zambia than currently understood; one study found a 32% prevalence of COVID-19 among a random sample of postmortem individuals that passed through the main capital city morgue in mid-2020, few of whom had been identified as SARS-CoV-2 positive premortem. Further, during peak transmission (the July 2020, January 2021, and June 2021 waves) SARS-CoV-2 was detected in 90% of postmortem patients.^13^

While the epidemic in Zambia was initially concentrated in the capital city, Lusaka, transmission has become more widespread. For Zambia, as in much of sub-Saharan Africa, potential superspreading events include church services held indoors or in tents with poor ventilation, funeral services, and other community gatherings. Using quantitative data collected from August 2020 through September 2021 among caregivers participating in an ongoing study, we explored attendance at superspreading events, as well as personal and perceived mask use among female caregivers in remote, rural Zambian communities.

## METHODS

### Study setting.

Zambia is a land-locked, lower-middle income country in southern Africa with a population of 18 million people, the majority (60%) of whom reside in rural areas.^14^ This analysis was conducted on data collected as part of a cluster-randomized controlled trial assessing the impact of community-based parenting groups on child development outcomes in Choma, Kalomo, and Pemba Districts of Southern Province and Nyimba District of Eastern Province (clinicaltrials.gov ID NCT03991182). These provinces each had 1.6 million people during the 2010 census, or 12% of the country population at the time.^15^^,^^16^ These provinces are primarily rural, with low population densities of 18.6 and 30.9 people per square kilometer in Southern and Eastern Provinces, respectively.^15^^,^^16^ Like much of sub-Saharan Africa, these provinces also have young populations, with nearly 50% of the population under age 15 and only approximately 3% of the populations over age 65. Zambia also has a high burden of infectious diseases including HIV/AIDS (11% prevalence among adults 15–59) and tuberculosis (455 cases per 100,000 population), making the population particularly vulnerable to COVID-19.^17^^–^^20^

The government of Zambia through the Ministry of Health and Zambia National Public Health Institute implemented an outbreak response to COVID-19 beginning March 13, 2020.^21^ Although implemented quickly and early in the pandemic, Zambia began easing COVID-19 restrictions on places of worship, businesses, restaurants, and bars in late April and early May 2020; schools, colleges, and universities in June 2020; and international arrivals in June 2020.^22^^–^^24^ Throughout the subsequent waves of infection, such restrictions were reenacted and rescinded accordingly.^25^ Through public broadcasting on television and radio, a call-in hotline, social media campaigns, and other community sensitization events, the government communicated its COVID-19 control strategy, mandates, and recommendations. All citizens, businesses, schools, and places of worship were expected to continue adhering to public health safety guidance including wearing of masks in public settings, social distancing when with individuals outside of one’s household; limiting size of gatherings; and washing of hands with soap and water or use of alcohol-based hand sanitizer. An exploratory qualitative study conducted in May and June 2020 found that Zambian respondents had increased odds of feeling at high risk of COVID-19 when compared with six other sub-Saharan African countries.^26^

### Study design.

In the catchment areas of 10 rural health centers, community-based parenting groups were rolled out in zones randomized to the intervention group.^27^ All primary female caregivers of children aged 0 to 5 years of age were invited to participate in an interactive, theater-based curriculum building the knowledge and skills of caregivers in the following areas of early childhood development stimulation, play, nutrition, and care-seeking.^27^ The groups were implemented at village-level with four to 10 caregivers registered per group.^27^ Each parenting group selected one of its members, known as the head mother, to be trained by community-based volunteers to facilitate each group session.^27^ Groups met approximately every other week, but were required by provincial health officials to stop meeting in February 2020 (before our study period). When parenting groups resumed in April 2020, they included messaging around COVID-19 transmission prevention and adherence to government guidelines. Throughout the study period, parenting groups adhered to the evolving government guidelines around COVID-19. In accordance with government guidelines, groups remained at or below 10 attendees, occurred outside, and sessions had handwashing stations supplied with soap and water. Participants were required to wear face masks and socially distance. All touch-based activities were removed from lesson plans after the emergence of COVID-19.

Within this overarching evaluation, as COVID-19 appeared and increased as a public health concern, we sought to understand how community risk perceptions around COVID-19 and their associated behaviors changed over time. Beginning in August 2020, the study began surveying caregivers attending group meetings on their attitudes and behaviors regarding COVID-19. Caregivers were asked each time they attended a group meeting about their perception of COVID-19 risk, the gatherings they had attended in the past 2 weeks, their own behaviors at these gatherings, and their perceptions of peers’ behaviors at these gatherings.

### Sampling.

The larger intervention took place in 10 health facility catchment areas (HFCAs) in Southern and Eastern Provinces and were purposively sampled based on prior participation in a maternity waiting homes intervention. These HFCAs were known to be equipped with community-based volunteers who were trained in maternal and child health. Each HFCA is further divided by the Zambia Ministry of Health into approximately 80 zones; 40 of these zones were randomized to the larger intervention. Within each intervention cluster, convenience sampling was used, and questionnaires were conducted among participating women. A sample size was not calculated a priori. All women were asked to complete a COVID-19 questionnaire during every community-based parenting group meeting that occurred between August 2020 and September 2021. Some women attended multiple meetings and therefore completed multiple questionnaires.

### Instrument design.

The COVID-19 questionnaires asked the respondent if they had attended gathering(s) since the last parenting group meeting, generally 2 weeks prior. The following kinds of gatherings were included: visits to the clinic, church, or funeral attendance and other gathering. Respondents were asked approximately how many other individuals were at the gathering, if the respondent wore a mask, if she perceived other attendees wore a mask (asked as a scale from no one to everyone), and where the gathering was located (indoors, outdoors, or in a tent). The questionnaire also asked respondents their perceived risk of catching COVID-19 (asked as scale from very low to very high). Demographic information for each respondent was collected separately within a parenting group attendance register. The study unique identifier for each respondent was included in the questionnaire. Caregivers often attended community-based parenting group meetings biweekly, and many caregivers submitted repeated questionnaires over time.

### Data collection methods.

Project staff trained community-based volunteers in completion of the COVID-19 Behaviors Questionnaire, who in turn trained parenting group head mothers. At each biweekly group meeting, the head mother administered a COVID-19 Behaviors Questionnaire to each respondent. Questionnaires were developed by project personnel and translated by a qualified translator into the local languages of Chitonga and Chinyanja. Questionnaires were completed on paper in the local languages. Project staff and short-term hires trained in research ethics continuously collected and extracted the paper questionnaires using SurveyCTO^®^ Collect Software installed on encrypted tablets. Demographic data from the first time each respondent attended the parenting groups were extracted.

Distance from the nearest government-assigned rural health center (RHC) was collected at the community-based parenting group (CBPG) and respondent-level. A village database was previously created by taking GPS coordinates in each village center within the study sites. ArcGIS (ESRI, Redlands, California, USA) was then used to calculate the distance from village center to the corresponding RHC.

When an individual completed the questionnaire more than once, they reported on different 2-week periods, and thus different gatherings attended. All gatherings, however, are not necessarily unique: two respondents, for example, may have attended the same funeral and would report their own perceptions and behaviors. Respondents were asked about four types of gatherings, and thus could report on four unique experiences in one questionnaire.

### Ethics.

This study was approved by the University of Zambia Biomedical Research Ethics Committee (Ref. No. 004-05-19) and the Boston University Medical Center Institutional Review Board (Ref. No. H-38950). Informed consent was waived by both institutional review boards for the COVID-19 questionnaire responses and demographic data due to the impracticality of consenting during parenting group meetings. Additionally, the National Health Research Authority; the Ministry of Health at the national, provincial, and district levels; and traditional chiefs in these districts granted official government approval to conduct the overarching study.

### Data analysis.

All quantitative analysis was conducted in SAS v9.4 (SAS Institute Inc., Cary, NC). Using the unique project identifiers, respondent names, and parenting group location information, the questionnaires for each respondent were linked to their demographic information.

**Figure 1.**
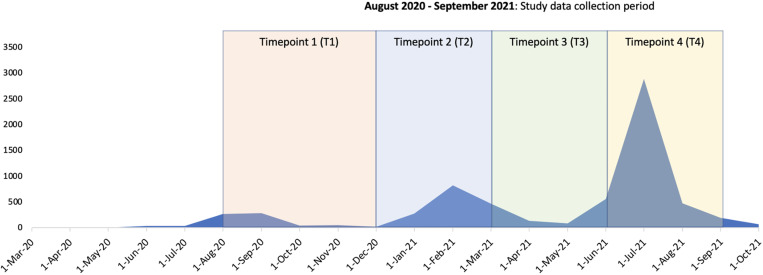
COVID-19 case trends, government policy, and guidance in Zambia between March 2020 and March 2021.

We created a timepoint variable, dividing the study period around the observed waves of COVID-19 Zambia. Timepoint 1 (T1) represents August to December 2020, as cases were rising in the country and before the first infection peak. Timepoint 2 (T2) represents January to March 2021, as the first peak in infections was recorded and eventually receded. Timepoint 3 (T3) represents a “recovery” period, when cases from the January peak receded, and there was little incidence of infection in Zambia. Timepoint 4 (T4) represents the second peak of cases between July and September of 2021 (Figure 1). Some caregivers completed multiple questionnaires, spanning each timepoint. In cases such as this, their demographic information contributes to each relevant timepoint.

We analyzed primary outcome variables including perceived risk of contracting COVID-19, attendance at community gatherings, perceived size of such gatherings, and mask wearing. We present frequencies and means for these outcomes. We ran logistic regressions to calculate crude and adjusted odds ratios (aOR). Adjusted models control for the following variables: gathering type, location, number of attendees, district, and timepoint, and where relevant they control for respondents’ demographic information.

We conducted sensitivity analysis to identify differences between linked and unlinked respondents: we conducted χ^2^ tests of independence and *t* tests to identify statistically significant differences in behaviors and demographics Supplemental Table 1).

## RESULTS

**Figure 2.**
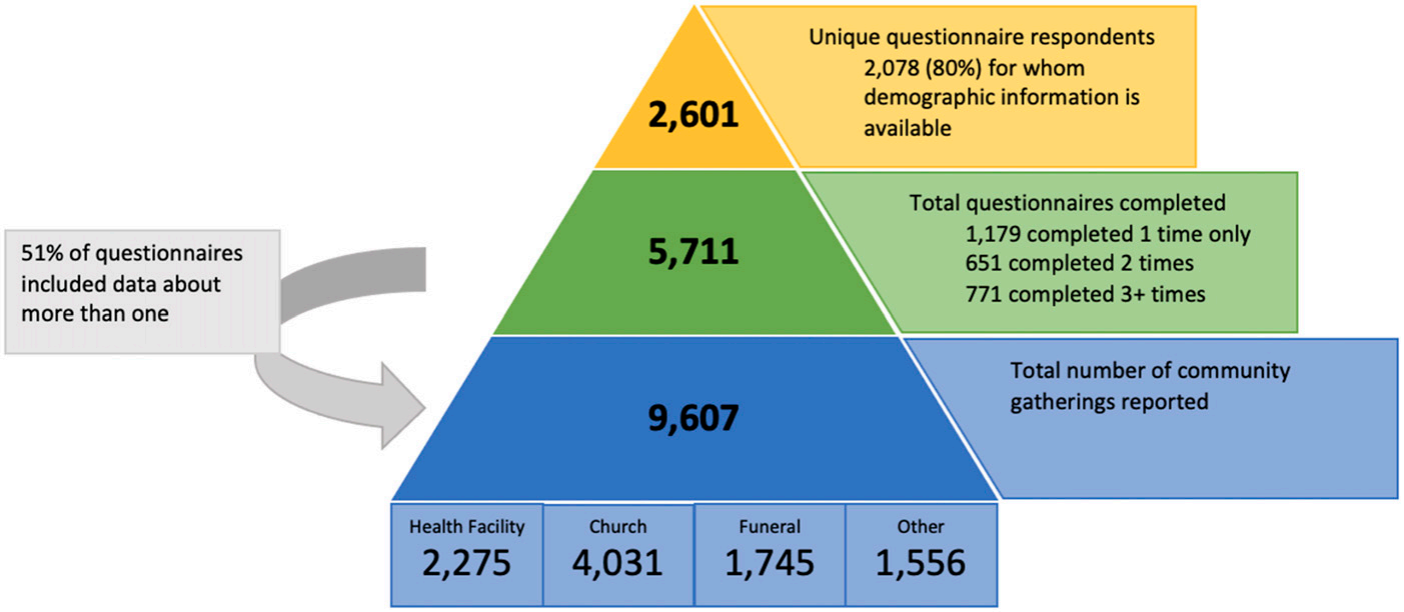
COVID-19 behaviors study sample sizes for respondents, questionnaires, and reported gatherings. Because of the nature of data collection, individual gatherings are not necessarily unique. Some respondents living in the same catchment areas may be reporting on the same events but from their own perspectives.

During our study period, 5,711 questionnaires were completed by 2,601 unique respondents: 1,179 individuals completed the questionnaire one time only; 651 completed the questionnaire twice; and 771 completed it three times or more (Figure 2). Respondents report their unique perceptions and behaviors at a total of 9,607 community gatherings: 42% were church gatherings, 24% clinic visits, 18% funerals, and 16% other.

Most respondents were women aged 15 to 34 (Table 1). Most were married, and a majority had completed primary education or higher. Attendance at community gatherings was generally high: 83% of respondents reported attending a community gathering in the 2 weeks before completing the questionnaire, with 51% attending more than one. When comparing timepoints, a slightly higher proportion of respondents reported attending gatherings in T1 (84.8%) than in the rest of the study period. Most respondents were from Southern Province (Choma, Pemba, and Kalomo districts). Most questionnaires were conducted during T1 (48.8%), with the fewest questionnaires conducted during T3 (3.9%).

**Table 1:** Demographic characteristics of COVID-19 behavior study respondents, at the questionnaire and respondent levels, overall and by timepoint

Questionnaire-level responses	All *N* = 5,711	T1 *N* = 2,788 (48.8%)	T2 *N* = 1,848 (32.4%)	T3 *N* = 221 (3.9%)	T4 *N* = 854 (14.9%)
District, *n* (%)					
Choma/Pemba	2,289 (40.1)	970 (34.9)	797 (43.1)	200 (90.5)	322 (37.7)
Kalomo	3,006 (52.6)	1,683 (60.4)	926 (50.1)	13 (5.9)	384 (45.0)
Nyimba	416 (7.3)	135 (4.8)	125 (6.8)	8 (3.6)	148 (17.3)
Distance from village to clinic, km, *n* (%)					
< 5	1,879 (32.9)	962 (34.5)	588 (31.8)	10 (4.5)	319 (37.3)
5.0–9.9	2,011 (35.2)	1,036 (37.1)	613 (33.2)	19 (8.6)	343 (40.2)
10–14.9	1,285 (22.5)	505 (18.1)	437 (23.6)	179 (81.0)	164 (19.2)
≥ 15	463 (8.1)	264 (9.5)	171 (2.1)	13 (5.9)	15 (1.8)
Reported attending a gathering in 2 weeks before questionnaire completion, *n* (%)					
Any gathering	4,796 (83.2)	2,364 (84.8)	1,517 (82.1)	177 (80.1)	688 (80.6)
Clinic	2,297 (39.9)	1,106 (39.7)	762 (41.2)	38 (17.2)	369 (43.2)
Church	4,073 (70.7)	2,050 (73.5)	1,292 (69.9)	160 (72.4)	529 (61.9)
Funeral	1,760 (30.5)	960 (34.4)	512 (27.7)	27 (12.2)	246 (28.8)
Other	1,575 (27.3)	873 (31.3)	387 (20.9)	30 (13.6)	266 (31.1)
Number of gatherings attended in 2 weeks before questionnaire completion, *n* (%)					
0	965 (16.9)	424 (15.2)	331 (17.9)	44 (19.9)	166 (19.4)
1	1,840 (32.2)	833 (29.9)	598 (32.4)	119 (53.8)	290 (34.0)
2	1,508 (26.4)	749 (26.9)	548 (29.6)	41 (18.5)	170 (19.9)
3	841 (14.7)	470 (16.9)	225 (12.2)	14 (6.3)	132 (15.5)
≥ 4	557 (9.7)	312 (11.2)	146 (7.9)	3 (1.4)	96 (11.2)

Respondent-level demographics*	All *N* = 2,078	T1 *N* = 1,300	T2 *N* = 1,093	T3 *N* = 103	T4 *N* = 688
Female, *n* (%)	2,025 (97.4)	1,268 (97.5)	1,066 (97.5)	103 (100)	667 (96.9)
Age, mean (SD)	28.2 (8.2)	27.9 (8.0)	28.7 (8.3)	26.0 (6.6)	28.2 (8.3)
Age categories, years, *n* (%)					
≤ 24	776 (37.3)	511 (39.3)	373 (34.1)	36 (34.9)	255 (37.1)
25–34	757 (36.4)	468 (36.0)	416 (38.1)	45 (43.7)	244 (35.5)
35–44	389 (18.7)	243 (18.7)	230 (21.0)	11 (10.7)	126 (18.3)
45–54	61 (2.9)	37 (2.8)	34 (3.1)	0 (0.0)	20 (2.9)
>55	10 (0.48)	3 (0.2)	6 (0.55)	0 (0.0)	4 (0.58)
Highest grade completed, mean (SD)	6.8 (2.8)	6.9 (2.8)	7.1 (2.5)	7.3 (2.6)	6.6 (3.0)
Education categories, *n* (%)					
No school	134 (6.4)	91 (7.0)	42 (3.8)	5 (4.9)	54 (7.8)
Some primary education (1–5)	334 (16.1)	201 (15.5)	164 (15.0)	13 (12.6)	112 (16.3)
Completed primary education/ some secondary (6–7)	732 (35.2)	436 (33.5)	394 (36.0)	34 (33.0)	252 (36.6)
Completed secondary education/more than secondary education (8+)	832 (40.0)	548 (42.2)	467 (42.7)	50 (48.5)	243 (35.3)
Marital status, *n* (%)					
Married, cohabiting	1,715 (82.5)	1,065 (81.9)	901 (82.4)	88 (85.4)	581 (84.4)
Divorced, widowed	200 (9.6)	111 (8.5)	100 (9.1)	11 (10.7)	66 (9.6)
Never married	125 (6.0)	103 (7.9)	73 (6.7)	4 (3.9)	35 (5.1)
Not indicated	38 (1.8)	21 (1.6)	19 (1.7)	0 (0.0)	6 (0.87)
District, *n* (%)					
Choma/Pemba	813 (39.1)	451 (34.7)	419 (38.3)	97 (94.2)	267 (38.8)
Kalomo	1,086 (52.3)	765 (58.8)	598 (54.7)	6 (5.8)	339 (49.3)
Nyimba	179 (8.6)	84 (6.5)	76 (7.0)	0 (0.0)	82 (11.9)

* Timepoint sample sizes sum to be greater than the total (*N* = 2,078) because of repeated questionnaires conducted with the same respondents. One respondent can contribute demographic data to more than one timepoint. 20% (*N* = 515) respondents could not be linked to demographic information because data were not yet available from parent study.

**Figure 3.**
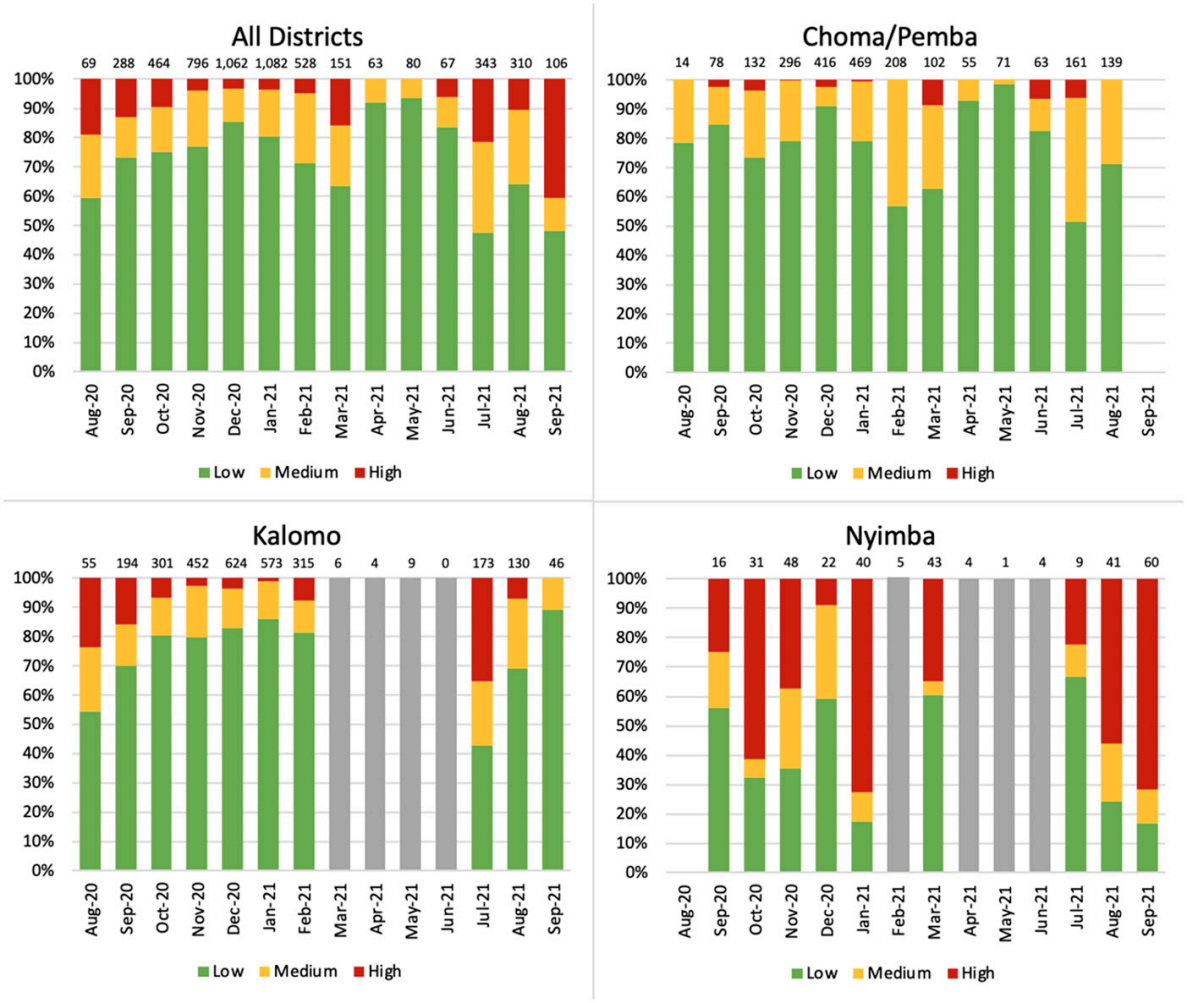
Respondents’ perceptions of risk of contracting COVID-19, stratified by district and timepoint. * Bars in gray represent months in which sample sizes were too small to present.

Generally, before the large wave of cases (T4) respondents’ perception of risk was low: 77.5% of respondents in T1, 72.8% of respondents in T2, and 85.5% of respondents in T3 reported feeling at low or very low risk of contracting COVID-19, compared with 51.5% in T4. Responses showed a general trend of increasing perceptions of risk from COVID-19 between T1 and T4 in all districts; this change over time was statistically significant (*P* < 0.0001). Districts in Southern Province (Choma, Pemba, and Kalomo) most frequently reported feeling at low risk of COVID-19. Conversely, more than 40% of respondents from Nyimba reported feeling at high risk over the entire study period (Figure 3).

Among respondents, the most frequently attended event type was church, with 71% attending a service in the 2 weeks before questionnaire completion (Table 2). Most respondents reported wearing a mask (80%) and perceived mask use among their peers (70%) at church services. The largest events were funerals, with a median of 200 attendees, but with high variance in numbers attending. Funerals ranged from 6 to 1,300 attendees. Only 30.6% of respondents reported attending a funeral, but for those who did, mask wearing was low for both respondents and their peers. Twenty-three percent of respondents who attended funerals reported that few to no other attendees were wearing masks. Funerals were also most often held outdoors. Other gathering types were the least attended events and had moderate mask use. These events included football games, community meetings, and weddings and varied in size and location.

**Table 2:** Attendance at large gatherings and practice of prevention behaviors, stratified by gathering type

	Type of gathering			
	Clinic	Church	Funeral	Other
Attended gathering in the past 2 weeks, *n* (%)	2,275 (39.8)	4,031 (70.6)	1,745 (30.6)	1,556 (27.2)
% attended gathering ≥ 50 people	380 (16.7)	2,538 (63.0)	1,514 (86.8)	672 (43.2)
Behavior of attendees: wore mask				
Self-reported mask wearing, *n* (%)	1,911 (84.0)	3,235 (80.3)	1,217 (69.7)	1,163 (74.7)
Perceived mask wearing among other attendees, n (%)				
Most/all attendees	1,571 (69.1)	2,821 (70.0)	988 (56.6)	959 (61.6)
Half of attendees	196 (8.6)	438 (10.9)	251 (14.4)	175 (11.2)
Few attendees	344 (15.1)	481 (11.9)	270 (15.5)	202 (13.0)
No attendees	42 (1.8)	149 (3.7)	134 (7.7)	101 (6.5)
Description of gathering				
Estimated number of attendees, median (IQR)	20 (26)	62 (60)	200 (250)	45 (70)
Perceived number of attendees, *n* (%)				
≤ 10	344 (15.1)	36 (0.9)	5 (0.3)	114 (7.3)
11–50	1,388 (61.0)	1,494 (37.1)	128 (7.3)	698 (44.9)
51–100	220 (9.7)	1,319 (32.7)	276 (15.8)	332 (21.3)
101–300	61 (2.7)	801 (19.9)	746 (42.8)	206 (13.2)
> 300	0 (0.0)	60 (1.5)	432 (24.8)	45 (2.9)
Respondent did not provide estimate	262 (11.5)	321 (8.0)	158 (9.1)	161 (10.3)
Location of gathering, *n* (%)				
Indoors	N/A*	2,990 (74.2)	179 (10.3)	245 (15.7)
Tent		104 (2.6)	89 (5.1)	82 (5.3)
Outdoors		792 (19.6)	1,388 (79.5)	1,144 (73.5)

IQR = interquartile range; N/A = not applicable.

* Although patients typically meet with their healthcare provider indoors, facilities have waiting spaces that are largely outdoors (or spaces with a roof but open walls) where most of the people sit while they wait. The indoor spaces are used by one to three patients at a time.

**Figure 4.**
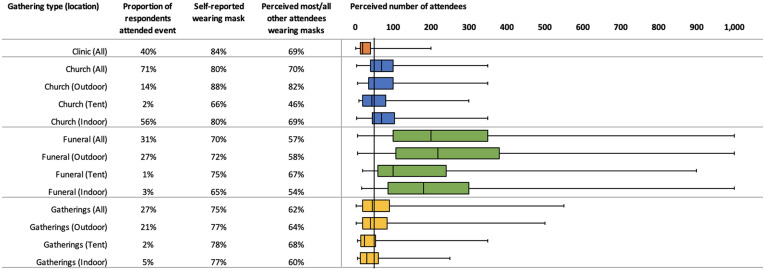
Self-reported mask use and perceived behaviors of other attendees by gathering type and location, including perceived number of attendees. * The vertical line at 50 perceived number of attendees serves to display the number of events that exceeded the government’s guideline of suggested gathering size.

At all gathering types, respondents self-reported wearing masks more frequently than they reported most or all their peers wearing masks (Figure 4). Many events, especially funerals, had wide ranges of perceived attendees, and were often larger than the 50 attendees recommended by Ministry of Health guidelines.^25^

After controlling for potential confounders in the dataset, respondents had lower odds of self-reported mask use and lower odds of mask wearing among other attendees at funerals compared with all other gathering types (self-use: aOR 0.44; other attendees: aOR 0.54; Table 3). Respondents also had the lowest odds of wearing masks (self-use aOR 0.50; or other attendees: aOR 0.45) at large events with more than 300 people, although most gathering sizes had lower odds of mask use compared with medium-sized events (11 to 50 attendees). Respondents in Nyimba District reported much lower odds of mask use by themselves or others at gatherings compared with Choma and Pemba (self-use aOR 0.26; other attendees aOR 0.29). Odds of mask use were highest in T4 compared with T1 (aOR 3.0).

**Table 3:** Questionnaire-level predictors of self and other attendee mask use at gatherings; bivariate models and multivariate regression model

	Respondent reported wearing mask			Respondent reported most/all other attendees wearing masks		
	Crude OR (95% CI)	Adjusted OR (95% CI)	*P* value	Crude OR (95% CI)	Adjusted OR (95% CI)	*P* value
Gathering type						
Clinic	Ref	Ref	< 0.0001	Ref	Ref	< 0.0001
Church	0.78 (0.7–0.9)	0.77 (0.7–0.9)		0.98 (0.9–1.1)	0.98 (0.9–1.1)	
Funeral	0.45 (0.4–0.5)	0.44 (0.3–0.6)		0.56 (0.5–0.6)	0.54 (0.4–0.7)	
Other	0.57 (0.5–0.7)	0.53 (0.5–0.6)		0.75 (0.6–0.9)	0.59 (0.5–0.7)	
Gathering location						
Indoors (including clinic)	Ref	Ref	0.09	Ref	Ref	0.0003
Outdoors/tent	0.78 (0.7–0.9)	1.1 (1.0–1.3)		0.89 (0.8–1.0)	1.2 (1.1–1.4)	
No. of attendees						
≤ 10	1.1 (0.8–1.4)	1.0 (0.8–1.4)	< 0.0001	0.95 (0.8–1.2)	0.95 (0.8–1.2)	< 0.0001
11–50	Ref	Ref		Ref	Ref	
51–100	0.44 (0.4–0.5)	0.59 (0.5–0.7)		0.67 (0.6–0.7)	0.72 (0.6–0.8)	
101–300	0.69 (0.6–0.8)	0.86 (0.7–1.0)		0.71 (0.6–0.8)	0.80 (0.7–0.9)	
> 300	0.39 (0.3–0.5)	0.50 (0.5–0.6)		0.43 (0.4–0.5)	0.45 (0.4–0.5)	
District						
Choma/Pemba	Ref	Ref	< 0.0001	Ref	Ref	< 0.0001
Kalomo	0.83 (0.7–0.9)	0.90 (0.8–1.0)		0.93 (0.8–1.0)	1.1 (1.0–1.2)	
Nyimba	0.28 (0.2–0.3)	0.26 (0.2–0.3)		0.35 (0.3–0.4)	0.29 (0.2–0.3)	
Timepoint						
T1	Ref	Ref	< 0.0001	Ref	Ref	< 0.0001
T2	1.6 (1.4–1.8)	1.6 (1.4–1.7)		1.6 (1.4–1.8)	1.6 (1.4–1.7)	
T3	1.4 (1.1–1.9)	1.1 (0.8–1.5)		1.4 (1.1–1.9)	1.1 (0.8–1.5)	
T4	2.6 (2.3–3.1)	3.0 (2.5–3.5)		2.6 (2.3–3.1)	3.0 (2.5–3.5)	

CI = confidence interval; OR = odds ratio.

At the individual level, among respondents with linked demographic data, older (35 years and older) respondents reported lower odds of mask wearing for themselves (aOR 0.85) or other attendees (aOR 0.80) compared with respondents aged 25 to 34 (Table 4). Additionally, low education level was associated with lower odds of mask use. As distance from the village to the clinic increased, odds of mask use decreased for respondents and perception of their peers.

**Table 4:** Individual-level demographic predictors of self and other attendee mask use at gatherings; bivariate models and multivariate regression model

Respondent characteristics	Self-reported mask wearing			Respondent reported other attendee mask wearing		
	OR (95% CI)	Adjusted OR (95% CI)	*P* value	OR (95% CI)	Adjusted OR (95% CI)	*P* value
Age, years						
15–24	0.99 (0.8–1.2)	1.0 (0.8–1.2)	0.13	1.0 (0.9–1.2)	1.1 (0.9–1.3)	0.018
25–34	Ref	Ref		Ref	Ref	
≥ 35	0.75 (0.6–0.9)	0.85 (0.7–1.0)		0.83 (0.7–1.0)	0.80 (0.7–1.0)	
Education						
None/some primary education	0.58 (0.5–0.7)	0.71 (0.6–0.9)	0.0006	0.76 (0.6–0.9)	0.86 (0.7–1.0)	0.084
Completed primary education/ Some secondary education	Ref	Ref		Ref	Ref	
Completed secondary education or more	1.2 (0.9–1.7)	1.0 (0.7–1.6)		1.2 (0.9–1.7)	1.2 (0.9–1.6)	
Married/cohabiting	0.68 (0.6–0.8)	0.83 (0.6–1.0)	0.10	1.1 (0.9–1.2)	0.87 (0.7–1.1)	0.23
Distance from village to clinic, km						
< 5	Ref	Ref	< 0.0001	Ref	Ref	< 0.0001
5–9.9	0.92 (0.8–1.1)	0.74 (0.6–0.9)		0.85 (0.7–1.0)	0.72 (0.6–0.9)	
10–14.9	0.85 (0.7–1.0)	0.70 (0.5–0.9)		0.75 (0.6–0.9)	0.64 (0.5–0.8)	
≥ 15	0.57 (0.5–0.7)	0.57 (0.4–0.7)		0.53 (0.4–0.7)	0.53 (0.4–0.7)	

CI = confidence interval; OR = odds ratio.

## DISCUSSION

We assessed perceived risk of contracting COVID-19, attendance at community gatherings, and adherence to the government recommended NPIs for COVID-19 among a sample of women in rural Zambia. Attendance at community events fluctuated over the timepoints, possibly in response to the waves of COVID-19 that overlayed T2 and T4. Although we found that odds of protective behaviors increased over those timepoints, individuals continued to gather and interact with their communities. Observed attendance to community gatherings was high over the course of our study period.

Mask wearing, both self-reported and perceived behaviors of others, was highest in clinics and church services. These gatherings, occurring in institutional settings, showed similar trends but are unique in the nature of the gatherings. Clinics and clinic staff have regulation over visitors, inherent knowledge about safety and health practices, and the greatest stake in the health and well-being of visitors. Thus, clinic visits had the highest proportion of respondents reporting wearing masks. Additionally, less than 2% of respondents reported that masks were not worn by peers at the clinic. Not surprisingly, the clinic infrastructure is conducive to enforcing NPIs for COVID-19 prevention. Qualitative data from the parent study suggests that facility-affiliated community health workers ensure that all patients wear masks upon entering the facility, handwashing stations are available, and social distancing measures are enforced while patients are awaiting treatment (Cite qual). Church services, a different type of institutional setting, display similarly high rates of self-reported mask use (80.8%). Mask wearing is a critical prevention measure in this context where churches are often crowded, poorly ventilated and attendees sing, releasing respiratory droplets and aerosols. Such settings have been reported to be high risk for disease transmission.^28^

Events including funerals and other community events (including weddings, community meetings, and football games) were less broadly attended by study respondents than the institutional gatherings. These events most often occurred outdoors but drew large crowds of attendees: 83% of funerals had more than 50 attendees, with 25% of respondents reporting event sizes greater than 300. Many community members attend, and often sing, talk, hug, and otherwise comfort the family of the deceased. Although only about a third of respondents attended funerals, more than 80% of those who attended reported that funerals had more than 50 attendees. With 10% of our study sample attending funerals of more than 350 other attendees, and reporting mask use ∼60%, there is a potentially high risk of COVID-19 transmission. The median size of other gatherings was lower (45), but some events also drew crowds of more than 500. Although 70% to 80% of these events were hosted outdoors, mask wearing was low, and social distancing data was not collected. Lower rates of mask use and higher attendance may be attributed to the informal nature of the events. In the absence of community leadership or an institution, there may be less enforcement of guidelines. A systematic review identified COVID-19 risk communication and community engagement plans in 13 African countries, and many strategies included leveraging community leaders, including community health workers and religious leaders. In the Democratic Republic of Congo, the country also leveraged community members including women leaders, civil society organization leaders, and young people.^29^ Our study did not detail the leadership present at each gathering type, but a further investigation into this could provide insight on community gatherings. Risk communication through community health workers and religious leaders would align with our analysis regarding institutional-based gatherings because there is inherent community leadership present in those settings; however, we do not know what type of other community mobilization efforts may have taken place at funerals, football games, weddings, and the like.

Respondent behaviors with regard to funerals proved to be risky. In Zambia, funerals are often large community-based events. In July 2021, South Africa limited funerals to 50 attendees and banned all after-funeral gatherings to reduce risk of spreading COVID-19.^30^ Other African countries, such as Zimbabwe, Ghana, and Botswana, have similarly imposed guidelines specific to funerals.^31^^–^^33^ In the absence of such regulation, funerals have potential to become superspreading events in Zambia as well. With increased mortality due to the COVID-19 pandemic, more families may be holding funerals, hosting loved ones and recent contacts of the deceased. This should be considered as variants continue to emerge, some with increasing transmissibility.

We found substantial differences in COVID-19 risk perception between the two provinces in which we conducted the questionnaires. In the three districts in Southern Province, risk perception was generally reported to be low, whereas in the study district in Eastern Province, Nyimba, perceived risk was much higher. Interestingly, in our regression models, respondents from Nyimba had reduced odds of wearing masks or seeing their peers wear masks, a notable contradiction to their reported risk perception. Nyimba contributed only 6% of the questionnaire data, so some of the differences may be attributed to sample size, but there are other factors to consider. Most cases of COVID-19 were centralized to Lusaka, but Southern Province reported some of the highest numbers of cases in the country in the early months of the pandemic.^34^ Perhaps higher case numbers and possible increased sensitization made community members more confident in their use of NPIs and the ability to protect themselves from contracting COVID-19. Additionally, country-wide reporting could have communicated high case numbers in urban areas, and low case numbers in rural areas (where our study was conducted), leading people to feel at lower risk. The study sites in Nyimba are situated near a main road, along which travelers frequently drive. Residents may feel that this puts them at higher risk of contracting COVID-19 but does not explain why their reported mask wearing would be lower. Additionally, COVID-19 health messaging may be different by district or leadership. In Zambia, sector partners worked to map densely populated areas and identify areas in need of more focus and sensitization efforts. The primary focus of this effort seemed to be centralized to Lusaka, but if other provinces were mapped in this way, it could lead to increased sensitization and fear for some people.^35^ A study conducted with Zambia’s district-level COVID-19 data from March to July 2020 suggested that environmental and socioeconomic factors such as population density, HIV prevalence, distance to towns, distance to airports may affect risk of COVID-19.^36^ It is conceivable that factors such as these (which cannot be analyzed in this study) do affect caseload and perception of risk in our study settings as well. These differences point to the nuanced ways in which communities can experience this pandemic differently.

The takeaways from this analysis are especially important as we consider the future of the COVID-19 pandemic. The Delta variant was first detected in Zambia in June 2021, and spread rapidly through the country, causing a third wave lasting through June and July 2021. The Delta variant was highly contagious, making safety precautions of vital importance. In November 2021, the Ministry of Health notified the public of the threat of the Omicron variant, preparing for a “possible eminent fourth wave.” Public health guidelines including surveillance, quarantining travelers, mask mandates, vaccination mandates for government and civil service workers, limiting church services, and limiting sizes of weddings and funerals were reinstated.^37^ Conversely, future vaccine availability may change many of these behaviors and perceptions of risk. During most of this study period, vaccines had not yet been distributed in Zambia. The first vaccine doses arrived in the country on April 14, 2021, with another shipment arriving in July 2021, but both supply and vaccinations rates were low. As of July 5, 2021, 142,000 people received their first dose of the vaccine, using half of the designated supply.^38^ As of June 30, 2022, 4.5 million individuals were fully vaccinated.^12^ Lessons learned through the COVID-19 pandemic may also have application to future infectious disease preparedness plans.

## LIMITATIONS

There are several limitations to this study. First, our analysis describes experiences from a very specific period of COVID-19 and may not be generalizable to subsequent waves or variants. Second, we surveyed only women who decided to attend CBPGs, during which they received health education, which often highlighted the importance of COVID-19 guidelines. This may skew responses toward adherence. Alternatively, sampling only those who chose to attend a community event, may result in a sample of individuals who perceive lower risk of contracting disease or may feel less risk averse. Other community members including women who may be eligible for the CBPGs but decide not to attend due to high perceived risk, men, and others outside parent study participation criteria may have different behaviors and perceptions. Third, self-reported responses may be subject to desirability bias. Questionnaires were administered by volunteers with whom women have developed a relationship. Similarly, perceptions of other gathering attendees are subjective: Caregivers were not trained on estimating numbers of other attendees, and we do not have observations to confirm behaviors or opinions. Fourth, we did not ask about social distancing, handwashing behaviors, or whether masks were worn snugly over the nose and mouth. We added these questions to later rounds of our instruments and may present them in future analyses. Fifth, due to the nature of the parent intervention, we do not have demographic information for all questionnaire respondents. Sixth, our study was conducted in rural areas of Zambia and may not be generalized to urban areas or other countries. Further, trends cannot necessarily be generalized to districts or regions within Zambia, as illustrated by the differences between Southern and Eastern Provinces.

## CONCLUSION

The results from this analysis, including the changing risk perception and mask use, suggest guideline dissemination penetrated the rural areas of Zambia. However, there is continuing need to optimize public health messaging to further encourage NPI use at funerals and other high-risk gatherings. The findings from this analysis should be considered as the COVID-19 pandemic continues to evolve. Lessons may also be applied to future pandemics or epidemics.

## Supplemental files


Supplemental materials
