# Supplementary material for: Lessons from a Year of COVID-19 in Zambia: Reported Attendance and Mask Wearing at Large Gatherings in Rural Communities
Source: Am J Trop Med Hyg. 2022 Dec 12;108(2):384–93. doi: 10.4269/ajtmh.22-0460 (PMC9896318; doi:10.4269/ajtmh.22-0460)
Supplement: Supplementary file 1 [file tpmd220460.SD1.pdf]

Supplementary Table 1. Sensitivity analysis of responses between questionnaires with and without demographic characteristics linked in the dataset

	**Questionnaires with demographic characteristics linked (N=4981)**	**Questionnaires without demographic characteristics (N=730)**	**P-value**
Attended gathering in past 2 weeks, n (%)	4,122 (82.8)	624 (85.5)	<0.0001
Perception of risk, n (%)			
High/very high	375 (7.5)	44 (6.0)	0.01
Medium	824 (16.5)	147 (20.1)	
Low/very low	3,628 (72.8)	507 (69.4)	
**Description of gathering**			
Clinic			
Attend, n (%)	1,965 (39.4)	310 (42.5)	0.12
Self-reported mask wearing, n (%)	1,641 (83.5)	270 (87.1)	0.22
Most/all other attendees mask wearing, n (%)	1,354 (68.9)	217 (70.0)	0.80
Estimated number of attendees, median (IQR)	20 (15, 40)	20 (11, 40)	-
Church			
Attend, n (%)	3,516 (70.6)	515 (70.5)	0.98
Self-reported mask wearing, n (%)	2,818 (80.1)	417 (81.0)	0.80
Most/all other attendees mask wearing, n (%)	2,460 (70.0)	361 (70.1)	0.77
Estimated number of attendees, median (IQR)	65 (40, 100)	60 (40, 100)	-
Funeral			
Attend, n (%)	1,522 (30.6)	223 (30.5)	0.99
Self-reported mask wearing, n (%)	1,050 (69.0)	167 (74.9)	0.10
Most/all other attendees mask wearing, n (%)	859 (56.4)	129 (57.8)	0.33
Estimated number of attendees, median (IQR)	200 (100, 360)	200 (101, 300)	
Other Gathering			
Attend, n (%)	1,340 (26.9)	216 (29.6)	0.13
Self-reported mask wearing, n (%)	982 (73.3)	181 (83.8)	0.0015
Most/all other attendees mask wearing, n (%)	828 (61.8)	131 (60.6)	0.85
Estimated number of attendees, median (IQR)	47.5 (20, 90)	40 (20, 90)	
